# Neurogenesis and Increase in Differentiated Neural Cell Survival via Phosphorylation of Akt1 after Fluoxetine Treatment of Stem Cells

**DOI:** 10.1155/2013/582526

**Published:** 2013-08-18

**Authors:** Anahita Rahmani, Danial Kheradmand, Peyman Keyhanvar, Alireza Shoae-Hassani, Amir Darbandi-Azar

**Affiliations:** ^1^Stem Cell and Tissue Engineering Department, Research Center for Science and Technology in Medicine (RCSTiM), Tehran University of Medical Sciences, Tehran, Iran; ^2^Faculty of Medicine, Islamic Azad University, Mashhad Branch, Mashhad 19988-96953, Iran; ^3^Medical Nanotechnology Department, School of Advanced Technologies in Medicine, Tehran University of Medical Sciences, Tehran, Iran; ^4^Rajaei Cardiovascular, Medical, and Research Centre, Iran University of Medical Sciences, P.O. Box 14185-615, Tehran, Iran

## Abstract

Fluoxetine (FLX) is a selective serotonin reuptake inhibitor (SSRI). Its action is possibly through an increase in neural cell survival. The mechanism of improved survival rate of neurons by FLX may relate to the overexpression of some kinases such as Akt protein. Akt1 (a serine/threonine kinase) plays a key role in the modulation of cell proliferation and survival. Our study evaluated the effects of FLX on mesenchymal stem cell (MSC) fate and Akt1 phosphorylation levels in MSCs. Evaluation tests included reverse transcriptase polymerase chain reaction, western blot, and immunocytochemistry assays. Nestin, MAP-2, and **β**-tubulin were detected after neurogenesis as neural markers. Ten **μ**M of FLX upregulated phosphorylation of Akt1 protein in induced hEnSC significantly. Also FLX did increase viability of these MSCs. Continuous FLX treatment after neurogenesis elevated the survival rate of differentiated neural cells probably by enhanced induction of Akt1 phosphorylation. This study addresses a novel role of FLX in neurogenesis and differentiated neural cell survival that may contribute to explaining the therapeutic action of fluoxetine in regenerative pharmacology.

## 1. Introduction

 Recent works in stem cell biology have opened up new ways in therapeutic strategies to replace lost cells with stem cells in injuries. Cell therapy will be an effective strategy for treating neurodegenerative diseases and spinal cord injuries [1]. However the important part of the successful therapy depends on differentiation and promoting the survival of implanted cells. The survival rate of stem cells after differentiation and transplantation is important for the efficacy of cell therapy [2]. Some factors are involved in the regulation of neurogenesis and their survival. Corticosteroids were the first hormones found to have a neurogenesis effect [3]. Neurotransmitters and growth factors also can affect neurogenesis [4]. Based on the fact that fluoxetine (FLX) improves brain function, researchers have explored the effect of antidepressants on the neurogenesis. FLX was the first selective serotonin reuptake inhibitor (SSRI) approved for the treatment of depression. On the molecular basis of its clinical efficacy, FLX increases serotonin synaptic availability [5]. Increased neurogenesis started by antidepressants in some manner contributes to their therapeutic effects [6]. These results provide some initial insights, but more work is needed to fully elucidate the SSRI actions on stem cell neurogenesis. 

 To promote extended survival of transplanted stem cells, we must modulate the properties of the cells. This purpose might be accomplished by overexpressing Akt1 protein which is a general mediator of cell survival signal. Akt is a serine/threonine kinase that plays a key role in the modulation of cell proliferation and survival. It is wellknown for its antiapoptotic effects against a variety of situations including oxidative and osmotic stress, irradiation, and ischemic shock [7–10].

 This study aimed to investigate whether fluoxetine could induce neurogenesis in mesenchymal stem cells and to further examine the putative role of Akt1 and its phosphorylation in FLX-mediated effects.

## 2. Materials and Methods

### 2.1. Isolation and Culture of Human Endometrial Stem Cells

This study was carried out in accordance with the Tehran University of Medical Sciences Ethical Committee Law. There was a consent form for each donor that is included in the supplementary data. Here we were using human endometrial stem cell (hEnSC) as a source of mesenchymal stem cell. Stem cell was obtained from 30 donors between 25 and 35 years old in the gynecology department as described previously [11]. Briefly, the biopsies from endometrium were dissected and then treated with collagenase (Sigma, USA) for 45 min. Isolated cells were suspended in phosphate buffered saline (PBS, Sigma, USA) supplemented with 10% fetal bovine serum (FBS; Gibco, UK), 0.2 mM EDTA (Sigma, USA), 100 U/mL penicillin, and 0.1 mg/mL streptomycin (Gibco, UK). The cells were layered on Ficoll Paque (Sigma, USA) and centrifuged at 400 g for 35 min at 4°C. EnSCs were retrieved from the buffy coat layer, washed in PBS, and then kept in DMEM/F12 medium (Gibco, UK). In the passage two, the CD146^+^, CD105^+^, and CD90^+^ cells were isolated from total cells [12] by the fluorescent analyzer cell sorter (FACS). 

### 2.2. Mesenchymal Stem Cells Differentiation

Human EnSCs were placed on collagen precoated plates with DMEM low-glucose medium. The cultures were kept in a humidified 10% CO_2_ atmosphere at 37°C for 10 days. The media were replaced with fresh media every 3 d. Stem cells that have grown to 70% confluence were pretreated with 1 *μ*M dimethyl-sulfoxide (DMSO, Sigma, USA) and then were treated with fluoxetine (1, 2, 5, and 10 *μ*M, Sigma, USA). Treatment with 1 *μ*M retinoic acid (RA, Sigma, USA) and dH_2_O was done as positive and negative controls in order. Also hEnSCs cultured in FLX treated media containing 10 *μ*M phosphatidylinositol-3-kinase (PI3-K) inhibitor (LY294002, Promega, USA) served as control cells to determine the Akt role. After treatment for 10 d, cells were subjected to examining the Akt1 phosphorylation by western blot and for specific markers of neural cells via reverse transcriptase PCR and immunocytochemistry assays. In each experiment, a nontreated group was tested.

### 2.3. Reverse Transcriptase PCR Analysis for Neural Specific Markers

For collection of total RNA from treated hEnSCs, we used an Isogen kit according to the manufacturer's instructions (Nippon Gene, Tokyo, Japan). RNA quantity and purity were determined by spectrophotometry (Beckman DU-65). Standard reverse transcription was performed using the AMV kit (Takara Biomedicals, Ohtsu, Japan) with 1 *μ*g RNA and 0.5 *μ*g oligo-dT per reaction, according to the manufacturer's instructions. Reaction mixtures included 2.5 *μ*L cDNA, 1x PCR buffer (AMS TM, Cinnagen, Iran), 200 *μ*M dNTPs, 0.5 *μ*M of each of forward and reverse primers (Table 1), and 1 U Taq DNA polymerase. Polymerase chain reactions were performed at 94°C for 1 min, 30 cycles 94°C for 30 s, 55–63°C for 30 s, and 72°C for 30 s, and 72°C for 10 min. Amplified DNA fragments were electrophoresed on 1.5% agarose gel. The gels were stained with ethidium bromide (10 *μ*g/mL) and photographed on a UV transilluminator (Uvidoc, UK).

### 2.4. Neural Markers Immunostaining

 The hEnSCs treated with FLX and FLX + 10 *μ*M PI3-K inhibitor (LY294002, Promega, USA) were fixed by incubation in 9% paraformaldehyde for 20 min and permeabilized with 0.5% Triton X-100 for 10 min as described previously [1]. The cells were then reacted with primary antibodies for Nestin (Sigma, USA), MAP-2 (Sigma, USA), *β*-tubulin-III (Chemicon, USA), and CD11b as a glial marker (Millipore, USA) at 4°C for 12 h, washed with PBS and reacted with the fluorescent isothiocyanate (FITC) conjugated secondary antibody (Sigma, USA) at room temperature for 2 h. Finally, the cells were washed with PBS three times, and DAPI was used for DNA staining. 

### 2.5. Detection of Akt1 Phosphorylation via Western Blotting

After FLX treatment, the cell lysates were collected and protein concentration was determined by using a protein assay kit (Bio-Rad, USA). Total cell extracts containing equal amounts of protein in sample buffer were subjected to WB analysis as described previously [13]. Briefly, the samples were boiled for 5 min and then separated by 12% sodium dodecyl sulfate polyacrylamide gel electrophoresis (SDS-PAGE). After that, the gel was transferred onto polyvinylidene difluoride (PVDF) membrane for blotting. The membrane was first blocked by incubation in bovine albumin at room temperature for 2 h and then incubated with anti-Akt1 antibody (Cell Signaling Technology, USA) and anti-phospho-Akt1 antibody (Cell Signaling Technology, USA) for 2 h at room temperature, washed for 3 times with tris buffer containing Tween-20 and incubated at room temperature with HRP conjugated secondary antibody for 2 h. The membrane was washed 5 times with tris buffer, and then specific bands were quantified using a ChemiImager System (Alpha Innotech Corporation). Anti-*β* actin antibody (Abcam, UK) was used as an internal control.

### 2.6. Viability Test (MTT) in Neural Differentiated Cells

 MTT (3-[4,5-dimethylthiazol-2-yl]-2,5-diphenyltetrazolium bromide) assay is a useful colorimetric test for detection of cell viability. MTT is for measuring the activity of cellular enzymes that reduce the yellow tetrazolium dye, to its insoluble formazan, giving a purple color. Differentiated mesenchymal stem cells were tested for their survival time in the presence or absence of FLX. The EnSCs were plated into 96 well enzyme linked immunosorbent assay (ELISA) plates, and the differentiation process was repeated as described previously in Section 2.2. After the differentiation for replacing the culture media, FLX was added to three of the wells and PBS was used as a negative control in three other wells. Culture plates were incubated for one week. During incubation (7 d), culture media were replaced with medium, containing FLX, FLX + LY294002, and PBS. Cell viability was assessed by MTT assay kit (Biotium, Hayward, USA). The MTT reagent (10 *μ*L) was added to the wells and incubated for 3 h. At the end of the incubation period, the medium was removed and 100 *μ*L DMSO was added into each well. To dissolve the formazan crystals, the supernatant was pipetted several times. Absorbance was measured on an ELISA plate reader at a wavelength of 540 nm.

### 2.7. Labeling Cell with 5-Bromo-20-deoxyuridine

 One *μ*M BrdU was added to the cultures for 6 h on the 8th day of FLX induction. The cells were observed on the 9th day of BrdU labeling and then fixed on the 10th day of development and stained for BrdU and Nestin with anti-BrdU monoclonal antibody (Sigma, St. Louis) overnight at 4°C and labeled with secondary antibody conjugated with Rhodamine for 120 minutes at room temperature. BrdU and Nestin positive cells were counted in 10 microscopic fields. The percentage and standard deviation of double stained cells among all Nestin positive cells were calculated.

### 2.8. Statistical Analyses

 Data shown were expressed as means ± S.D. from data obtained in three independent experiments. MTT and western blot results are obtained from six independent experiments. ANOVA was used to compare the effects of all treatments. Differences of viability within experimental groups were determined by the Mann-Whitney test. Differences were considered statistically significant at *P* ≤ 0.05.

## 3. Results

### 3.1. Human Endometrial Stem Cell Culture

**Figure 1:**
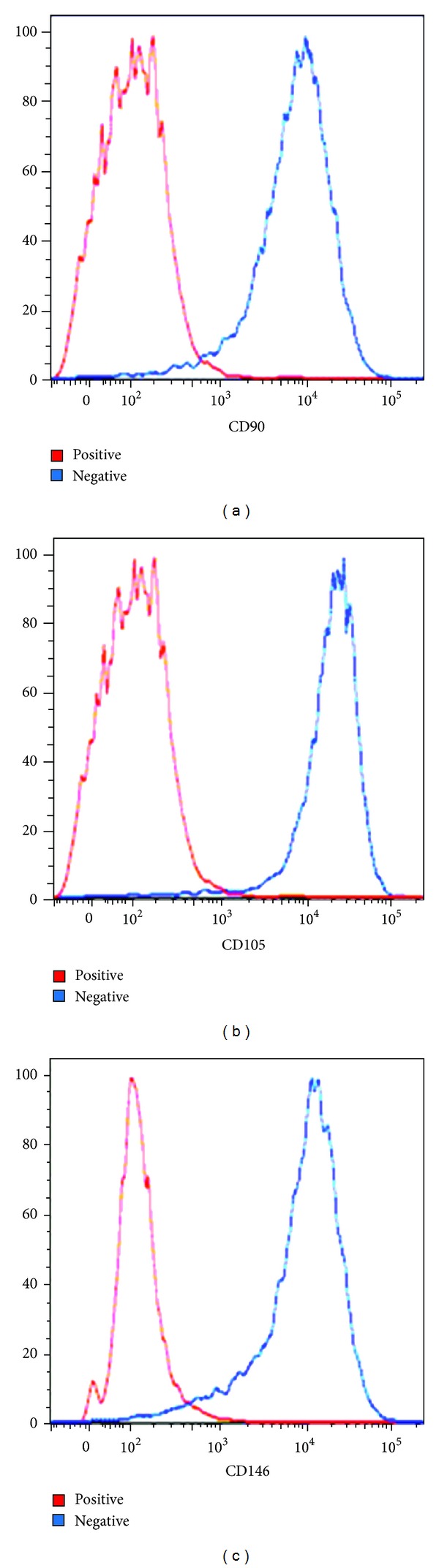
Characterization of human endometrial stem cells via fluorescent analyzer cell sorter (FACS). Human EnSCs after the second passage were sorted and analyzed by FACS for CD105^+^, CD146^+^, and CD90^+^ markers.

**Figure 2:**
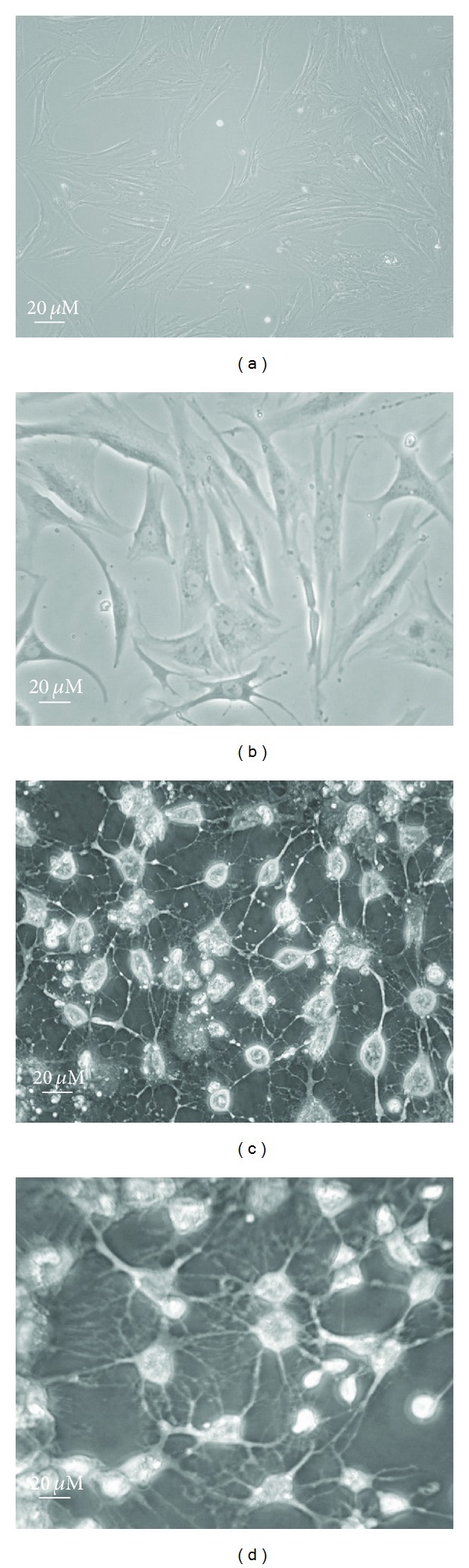
Morphological characteristics and differentiation of hEnSCs into neural cells. (a) Human EnSC (passage 2) in DMEM low glucose (b), after 10 d in DMEM pretreated with DMSO (c), plus 10 *μ*M of FLX and (d) 1 *μ*g/mL of retinoic acid as a positive control (magnification: ×400).

 Human EnSCs analysis by FACS showed that more than 90% of the cells were CD146+, CD105+, and CD90+ (Figure 1). This test confirms the true isolation of stem cells from the lining womb. To determine whether the hEnSCs could respond to FLX or not, we treated the stem cells with 1–10 *μ*g serial concentrations of FLX over a period of 10 d on collagen precoated and DMSO pretreated plates. In this work, human EnSCs with spindle shaped morphology were cultured (Figure 2(a)). After 10 d, fibroblast-like cells with spindle-shape morphology appeared on culture dishes (Figure 2(b)). Considering the role of FLX, we investigated its effect on the stem cell differentiation. Dendrite and axon formation and also neurite outgrowth that are phenotypic changes into neural fate were obvious (Figure 2(c)), and it was comparable with retinoic acid effects as a positive control (Figure 2(d)).

### 3.2. Expression of Differential Neural Genes

**Figure 3:**
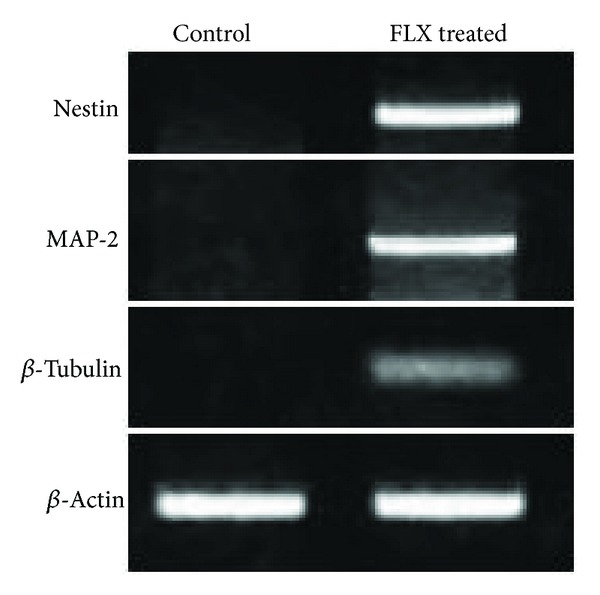
Expression of differential genes. Expression of *Nestin*, *Map-2*, and **β*-tubulin *neuron markers was analyzed in control culture and FLX (10 *μ*M) treated and induced differentiation culture after 10 d.

 The *in vitro *study is conducted to determine that the neurogenesis regulating effect could be replicated in cell culture system. One week after the treatment of hEnSCs, we examined the neural markers by RT-PCR. RT-PCR analysis was indicative of the expression of *nestin*, *map-2*, and **β*-tubulin* genes as shown in Figure 3. 

### 3.3. Immunostaining of Neuronal Induced Specific Markers

**Figure 4:**
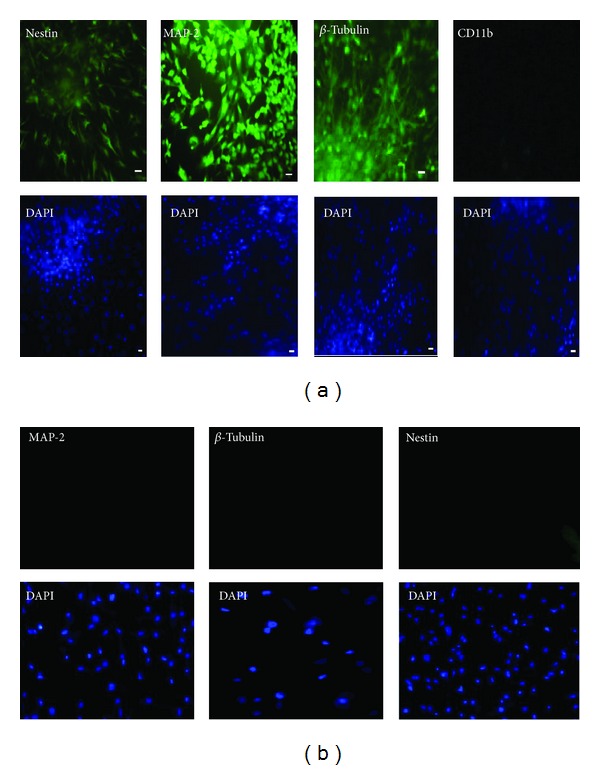
Immunostaining of hEnSCs differential markers. Expression of neuron markers including Nestin, MAP-2 and *β*-tubulin was analyzed in FLX induced differentiation after 10 d. The expression of CD11b as a glial marker was not obvious. The size bar is 10 *μ*m (a). Immunostaining of LY294002 treated hEnSCs after culture in FLX supplemented medium. There is not an expression of neuron markers in FLX induced differentiation after 10 d (b).

Fluoxetine treated and differentiated mesenchymal stem cells were visualized by the staining of some neuronal markers. Immunocytochemistry was used to analyze the Nestin, MAP-2, and *β*-tubulin proteins. The result showed that these proteins were detected after treatment in 10 d (Figure 4(a)). Also a group of cells that were treated with LY294002 as a phosphatidylinositol-3-kinase (PI3-K) inhibitor did not express the neural markers even after 10 d treatment by FLX (Figure 4(b)).

### 3.4. Overexpression of Phosphorylated Akt1

**Figure 5:**
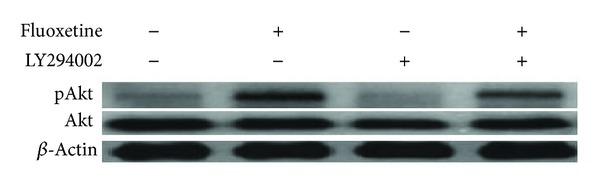
Western blot results after treatment of hEnSC with 10 *μ*M fluoxetine after 10 d. Protein samples (100 *μ*g) were loaded onto SDS PAGE gels and transferred to PVDF membranes. The induced EnSCs produced an increase expression of phosphorylated Akt1 protein. *β*-Actin was used as an internal control.

 Phosphorylation of Akt1 in FLX-treated mesenchymal stem cells as detected by western blot was higher than non-FLX-treated stem cells. Also LY294002 as a PI3-K inhibitor inhibited the phosphorylation of Akt1 significantly (Figure 5).

### 3.5. Viability of FLX Treated Cells

**Figure 6:**
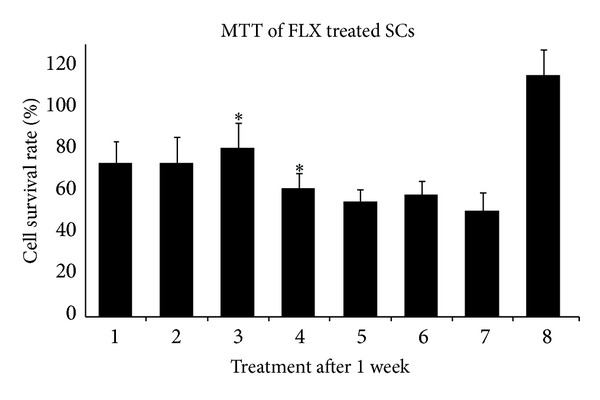
MTT shows EnSCs differentiated neural cell viability rate in the presence of FLX, FLX + LY294002, LY294002, and retinoic acid after one week (*P* ≤ 0.05). The numbers from the left side are as follows: (1) 1 *μ*M FLX, (2) 5 *μ*M FLX, (3) 10 *μ*M FLX, (4) 10 *μ*M FLX + 10 *μ*M LY294002, (5) 10 *μ*M LY294002, (6) 1 *μ*M retinoic acid, (7) neural differentiated cells without FLX supplement, and (8) undifferentiated mesenchymal stem cells.

**Figure 7:**
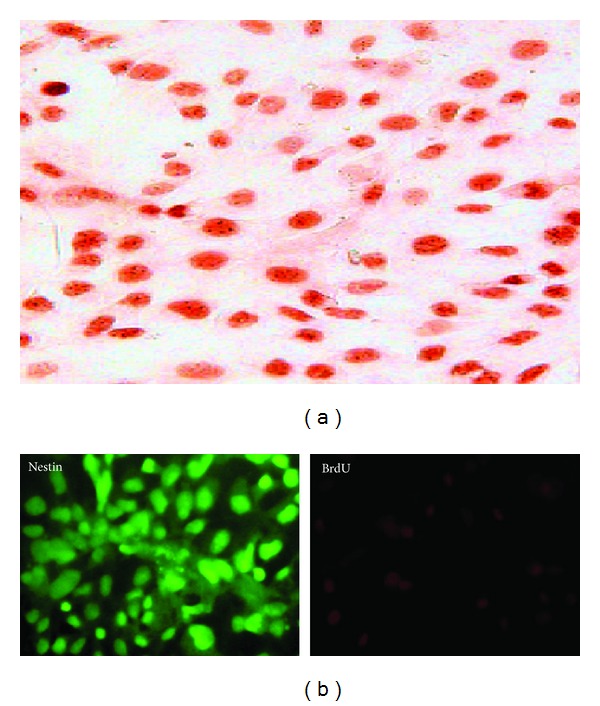
Endometrial stem cells labeled by BrdU (a). Cultures were treated with FLX and labeled with BrdU. They were then stained with antibodies against BrdU and nestin, followed by either rhodamine- (for BrdU, red) or FITC- (nestin, green) labeled secondary antibodies (b). Data are representative of 3 independent experiments.

 The effect of FLX on the survival period of the differentiated stem cells was observed directly and investigated by MTT assay. As shown in Figure 6, FLX treatment affects the cell survival rate significantly (*P* < 0.05) higher than all other groups. While the retinoic acid strongly suppressed cell survival in this assay, the suppressive effect of FLX on differentiated stem cells was very low. The continuance of FLX treatment extended the time and percentage of cell viability (Figure 6). The BrdU labeled cells were observed 24 h after labeling that showed over 90% of hEnSCs were BrdU positive (Figure 7(a)). The amounts of Nestin-BrdU positive cells were determined on the 10th day in cultures. The percentage of double stained cells among all Nestin positive cells and standard deviation were calculated from data of 10 microscopic fields. Cultures treated with BrdU and fixed on the 10th day contained about 60% double stained positive cells (Figure 7(b)).

## 4. Discussion

 Differentiation of mesenchymal stem cells into a specific lineage, their survival, and functionality are related to molecular regulation. The main finding of this research is that the fluoxetine antidepressant could induce neurogenesis in mesenchymal stem cells and upregulate the phosphorylation of serine/threonine kinase Akt1, and therefore it could be a differentiation and proliferation signaling mediator in cell therapy. 

 Selective serotonin reuptake inhibitors like FLX facilitate signaling of serotonin by inhibiting its reuptake. At the molecular levels, FLX increases synaptic availability of serotonin [14]. It has been shown that FLX could inhibit or activate PI3k/Akt or ERK1/2 pathways depending on the cell types and their function [15–17]. So it could have a dual role in the cancer therapy and cell therapy in regenerative medicine. For this purpose, we selected the Akt signaling to determine its role in mesenchymal stem cell proliferation, differentiation, and survival. 

 First, our *in vitro *study was conducted to determine the possibility of neurogenesis by FLX in the mesenchymal stem cell system (Figures 2(a) and 2(b)). Our results demonstrated that hEnSCs could be induced to differentiate into neurons in the presence of FLX (Figure 2(c)) as was seen in cells treated with retinoic acid (Figure 2(d)). High concentrations of FLX have nonserotoninergic effects on cells [5]. Nonserotoninergic targets of fluoxetine that mediate its other effects are likely to have a lower binding affinity or lower availability for FLX. Inhibition of cytochrome P450 is known to be due to a nonserotoninergic effect of FLX [18]. Lee et al. (2008) showed that liver cytochrome P450-dependent ROS formation is responsible for cyclin-A downregulation and inhibition of neural progenitor cell proliferation [19] so inhibition of cytochrome P450 may inhibit cyclin-A downregulation and restoring cyclin-A reverse inhibition of neural progenitor cell proliferation. However, this induction did not occur in the brain and suggested a tissue-specific response [20]. It has been demonstrated that the SSRIs interact with the *eag *voltage-gated K_1_ channel [21], and voltage-gated potassium channels play a functional role in the development of human neural progenitor cells and differentiation of neurons [22]. Also studies by de Oliveira and colleagues in 2012 showed that *eag* potassium channel expression in the rat hippocampus has been associated with neural cell survival and transient brain ischemia [23]. 

 In our experiment, fluoxetine-induced hEnSCs displayed neuronal morphology with axon formation and neurite outgrowth and expressed Nestin and other neuronal specific molecular markers by the reverse transcriptase polymerase chain reaction (Figure 3) and immunocytochemistry (Figure 4(a)) assays. EnSCs cultured in serum-free DMEM/F12 medium could not differentiate or even proliferated constantly. These findings indicate FLX-induced EnSCs may provide a stem-cell-based way to generate neuronal cells for the treatment of neurodegenerative diseases. This finding could determine that the endometrial stem cells express serotonin receptors. In neuropharmacology, it may be beneficial to study the effect of the different antidepressants on the neurogenesis since this provides insights into the regulation of stem cell proliferation. 

 Regeneration of neurons that undergo cell death soon after injury is the main goal of all cell-based therapies. From the point that neurogenesis has the capacity to enhance brain repair and/or spinal cord repair after ischemia and/or trauma by replacement of dead cells or seriously injured cells FLX neurogenesis could lead to recovery of these cells. FLX would appear to be a candidate as a neurogenesis factor. Our experiment showed that the hEnSCs expressed neural cell markers after FLX treatment, but there was no expression of CD11b marker that is a specific glial marker (Figure 4(a)). This is an important point because glial scar formation that occurs following CNS injuries is an important obstacle of neuroregeneration. FLX could introduce a regenerative product in neuronal injuries and find its position in regenerative pharmacology.

 Many groups have examined the potential of neuron replacement from embryonic stem cells [24], induced pluripotent stem cells (iPS) [25, 26], and cord blood derived stem cells. Endometrial stem cells have demonstrated superior potential. They expand rapidly and they have fewer technical and ethical problems so they have great potential as therapeutic agents and autologous grafts [2]. Our team has previously shown that the endometrial stem cells could be differentiated successfully into other lineages of cells especially neural cells [1, 11]. It represents a progress in the development of a new source of neurons. 

 We further confirmed the protective mechanisms of Akt1 phosphorylation as a survival signaling that introduces this pathway as a potential target for stem cell survival after differentiation of mesenchymal stem cells (Figure 6). PI3k/Akt is the important cell survival pathways and phosphorylation of Akt mediates anti-apoptosis in many kinds of cells [27]. In 2003, Kim et al. have shown that the Akt activation results in phosphorylation and then inactivation of the pro-apoptotic protein glycogen synthase kinase 3 (GSK3b), thus extending the cell survival period [28]. In support of the role of Akt1 phosphorylation, we found that the PI3K inhibitor LY294002 in the concentration of 10 *μ*M decreased the FLX mediated Akt phosphorylation (Figure 5), differentiation (Figure 4(b)), and the viability of neuronal differentiated cells (Figure 6). This suggests a key role for activating Akt1 in the protection of mesenchymal stem cell from the precocious death. Also we have labeled the hEnSCs with BrdU to obtain more exact cell proliferation rate (Figure 7(a)). The FLX treated stem cells that were labeled with BrdU showed double-staining for both BrdU and Nestin on the 10th day. Cells acquiring Nestin positive phenotype seemed to go through cell cycles enough to dilute the BrdU label to the non-detectable levels. In our experiment, BrdU was added on the 8th day of induction, and about half of nestin positive cells carried BrdU-label on the 10th day (Figure 7(b)). In cultures that FLX was absent about 70% of Nestin positive cells carried BrdU-label.

 In 2006, Frebel and Wiese confirmed the neuronal survival and differentiation implicated PI3-K/AKT in synaptic plasticity, learning, and memory in the mammalian brain [29].

 Although this finding supports the neurogenesis role of SSRIs, it has been suggested that excessive neurogenesis is not beneficial and could result in inappropriate migration into existing neural networks. Scharfman and Hen believe that this manner could cause some pathological conditions as epilepsy [30], but previously Rush et al. reporting that SSRIs could lose their effect after long-term use [31]. This has been called relapse during maintenance treatment. A possible mechanism underlying this effect is the weakness of neurogenesis because of excessive stimulation. So it is possible that long-term SSRI therapy might affect PI3/Akt pathway and its activation that leads to cognitive functions by disrupting the normal regulation of neurogenesis.

## 5. Conclusion

 In conclusion our data argue with fluoxetine effect on mesenchymal stem cell neurogenesis and its role in the phosphorylation of Akt1 in the differentiated cells to increase the survival rate of differentiated cells.

## Figures and Tables

**Table 1:** Primers used for specific neuronal genes expression.

Target genes	Primer sequences	Accession number
Nestin	F: 5′-GCCCTGACCACTCCAGTTTA-3′	NM:051373
	R: 5′-GGAGTCCTGGATTTCCTTCC-3′	
Map-2	F: 5′-CCATTTGCAACAGGAAGACAC-3′	NM:002374.3
	R: 5′-CAGCTCAAATGCTTTGCAACTAT-3′	
*Β*-tubulin	F: 5′-ATGTACGAAGACGACGAGGAG-3′	NM:BC:003021
	R: 5′-GTATCCCCGAAAATATAAACACA-3′	
*β*-actin	F: 5′-AAGAGAGGCATCCTGACCCT-3′
	R: 5′-ACATGGCTGGGGTGTTGAAGC-3′
